# Associations between Dietary Iron and Zinc Intakes, and between Biochemical Iron and Zinc Status in Women

**DOI:** 10.3390/nu7042983

**Published:** 2015-04-20

**Authors:** Karen Lim, Alison Booth, Ewa A. Szymlek-Gay, Rosalind S. Gibson, Karl B. Bailey, David Irving, Caryl Nowson, Lynn Riddell

**Affiliations:** 1 Centre for Physical Activity and Nutrition Research, School of Exercise and Nutrition, Deakin University, 221 Burwood Highway, Burwood, Victoria 3125, Australia; E-Mails: k.lim@deakin.edu.au (K.L.); alison.booth@deakin.edu.au (A.B.); ewa.szymlekgay@deakin.edu.au (E.A.S.-G.); caryl.nowson@deakin.edu.au (C.N.); 2 Department of Human Nutrition, University of Otago, PO Box 56, Dunedin 9054, New Zealand; E-Mails: rosalind.gibson@otago.ac.nz (R.S.G.); karl.bailey@otago.ac.nz (K.B.B.); 3 Australian Red Cross Blood Service, 17 O’Riordan Street, Alexandria, New South Wales 2015, Australia; E-Mail: dirving@redcrossblood.org.au; 4 University of Technology, Sydney 15 Broadway, Ultimo, New South Wales 2007, Australia

**Keywords:** iron, zinc, women, minerals, nutritional status

## Abstract

Iron and zinc are found in similar foods and absorption of both may be affected by food compounds, thus biochemical iron and zinc status may be related. This cross-sectional study aimed to: (1) describe dietary intakes and biochemical status of iron and zinc; (2) investigate associations between dietary iron and zinc intakes; and (3) investigate associations between biochemical iron and zinc status in a sample of premenopausal women aged 18–50 years who were recruited in Melbourne and Sydney, Australia. Usual dietary intakes were assessed using a 154-item food frequency questionnaire (*n* = 379). Iron status was assessed using serum ferritin and hemoglobin, zinc status using serum zinc (standardized to 08:00 collection), and presence of infection/inflammation using C-reactive protein (*n* = 326). Associations were explored using multiple regression and logistic regression. Mean (SD) iron and zinc intakes were 10.5 (3.5) mg/day and 9.3 (3.8) mg/day, respectively. Median (interquartile range) serum ferritin was 22 (12–38) μg/L and mean serum zinc concentrations (SD) were 12.6 (1.7) μmol/L in fasting samples and 11.8 (2.0) μmol/L in nonfasting samples. For each 1 mg/day increase in dietary iron intake, zinc intake increased by 0.4 mg/day. Each 1 μmol/L increase in serum zinc corresponded to a 6% increase in serum ferritin, however women with low serum zinc concentration (AM fasting < 10.7 μmol/L; AM nonfasting < 10.1 μmol/L) were not at increased risk of depleted iron stores (serum ferritin <15 μg/L; *p* = 0.340). Positive associations were observed between dietary iron and zinc intakes, and between iron and zinc status, however interpreting serum ferritin concentrations was not a useful proxy for estimating the likelihood of low serum zinc concentrations and women with depleted iron stores were not at increased risk of impaired zinc status in this cohort.

## 1. Introduction

The 2011–2012 Australian Health Survey indicated that 12% of women 16–44 years of age had depleted iron stores (serum ferritin < 15 μg/L [1]) compared to 1%–2% of Australian men [2]. Premenopausal women with iron stores at this level are more likely to experience impaired physical performance [3], and there is some evidence that cognitive ability may also be negatively affected [4,5]. As iron is found in similar food sources to zinc [6,7], inadequate dietary iron and zinc intakes may occur simultaneously [8]. There is also evidence that the form of iron and zinc in plant-based foods is less readily available for absorption than from diets containing meat, perhaps more so than other trace minerals [9,10]. It is widely acknowledged that the inorganic form of iron, non-heme iron, is less bioavailable than heme iron [11], and it has been hypothesized that analogous to non-heme iron, inorganic zinc salts in plants are a less bioavailable form of dietary zinc [12,13]. Moreover, compounds such as phytate may further impede absorption of both iron and zinc from foods [9,10]. Due to these diet-based similarities, there is potential for iron and zinc status to be positively correlated [14,15,16,17], however there are no data on zinc status to explore this relationship in Australian women. Assessment of zinc status is notoriously difficult [18], however as zinc plays a crucial role in immune function [19] and may be linked to depression [20], assessing zinc status to ensure optimal nutrition is important to health. Serum zinc, the most commonly used biomarker of zinc status, undergoes diurnal variation and is reduced following a meal [21]. As circulating zinc is present in such low concentrations, contamination of serum samples is a concern, and trace element-free equipment is required [21]. Given the challenges associated with measuring zinc status, understanding the relationship between biochemical measures of iron and zinc may provide an opportunity to use iron status as a proxy to screen women to assess their risk of impaired zinc status. Three studies in premenopausal women conducted in industrialized countries have reported correlations between serum ferritin and serum zinc concentrations ranging from 0.10 to 0.45 [14,16,17], and one has found significantly lower serum ferritin concentration in women with low zinc status (mean serum ferritin 10.8 (SD 7.2) μg/L *vs.* 26.0 (SD 15.3) μg/L) [15]. These findings indicate there may be an association between iron and zinc status, however only one study [17] accounted for infection and inflammation, which are known to elevate serum ferritin and suppress serum zinc concentrations [1,21]. Investigating the association between iron and zinc status whilst accounting for inflammation may further our understanding of the relationship between these two nutrients and provide some insight into whether depleted iron stores may predict risk of impaired zinc status among Australian women.

Our aims for this study were to: (1) describe dietary intakes and biochemical status of iron and zinc; (2) investigate associations between dietary iron and zinc intakes; and (3) investigate associations between biochemical iron and zinc status in a sample of premenopausal women aged 18–50 years in Australia.

## 2. Methods

### 2.1. Study Design

Women 18–50 years of age were recruited for this cross-sectional study in three phases: (a) from the student and staff population at Deakin University (Burwood, Melbourne, Australia) between July and November 2010 (*n* = 51); (b) from blood donors registered with the Australian Red Cross Blood Service (Blood Service; Sydney, Australia) between November 2010 and May 2011 (*n* = 203); and (c) from the student and staff population at Deakin University (Burwood, Melbourne, Australia) and residents of metropolitan Melbourne between July 2012 and January 2014 (*n* = 142). Written informed consent was obtained from all participants and ethical approval was granted by the Deakin University Human Research Ethics Committee (references 2009-191 and 2012-046) and the Blood Service Ethics Committee (reference 2010#01).

### 2.2. Recruitment and Eligibility

Recruitment at the Deakin University Burwood campus was conducted via flyers, announcements in lectures, and on the university’s online learning portal. Recruitment at the Blood Service was conducted as part of routine phone calls reminding registered blood donors when they are eligible to make their next blood donation. Recruitment of Melbourne residents was conducted via Facebook (‘sharing’ of a Facebook page about the study and paid advertisements of this page within Facebook) and printed advertisements in local newspapers.

Across all recruitment phases, women who had been through menopause, who were currently pregnant or lactating, or had been in the past 6 months, were ineligible to participate. In the recruitment period from 2012 to 2014, we introduced a criterion to exclude women who had donated blood in the previous 12 months as a number of blood donors had been recruited from 2010 to 2011.

### 2.3. Measures

For Melbourne-based recruitment, data was collected at the Deakin University Burwood campus. For Blood Service recruitment, data was collected at the Blood Service collection center when the women presented to make their scheduled blood donation. Data collection involved paper-based questionnaires and a blood sample.

#### 2.3.1. Anthropometric, Demographic, and Blood Donation Characteristics

Participants recruited in Melbourne had height and weight directly measured. Height was measured to 0.1 cm using a wall-mounted stadiometer and weight measured to 0.1 kg using electronic scales. Women recruited at the Blood Service self-reported height via questionnaire and had weight measured to 1 kg as per the Blood Service standard practice. Body mass index (BMI) was calculated as kg/m^2^ and interpreted using World Health Organization cut-offs for adults, with normal range being 18.5 to 24.9 kg/m^2^, overweight being 25.0 to 29.9 kg/m^2^, and obese being > 30 kg/m^2^ [22]. All participants completed a paper-based questionnaire to report their age, education level, cigarette smoking status, and use of oral contraception (protective against impaired iron status [23] and associated with suppressed serum zinc concentration [21]).

Women recruited in Melbourne in 2010 self-reported blood donor status and frequency of donation via questionnaire. Blood donation history of women recruited at the Blood Service was sourced from Blood Service records. From this information, women were dichotomized as having/not having made a donation in the previous 12 months. The amount of blood donated was quantified using conversion factors. Each whole blood donation was considered to be 470 mL and each apheresis (plasmapheresis and plateletpheresis) donation was considered to be 60 mL of whole blood. Blood donation was also considered as a continuous variable of mL donated in the past year.

#### 2.3.2. Dietary Intake

Participants’ usual dietary intakes were recorded using the Cancer Council Victoria Dietary Questionnaire for Epidemiological Studies v3.1 (DQES v3.1), a paper-based semiquantitative 154-item food frequency questionnaire (FFQ) that assesses the consumption frequency of 140 foods and beverages over the previous 12 months. The previous version of this FFQ [24] has been validated against seven-day weighed food records, with Bland Altman analyses finding mean difference of −0.22 mg/day and 95% CI limits of agreement of −8.1 to 7.7 for dietary iron intake, and mean difference of −0.82 and 95% CI limits of agreement of −7.2 to 5.6 for dietary zinc intake [25]. Pearson correlation coefficients of intakes from the food records and DQES v2 was *r* = 0.44 for dietary iron and *r* = 0.40 for dietary zinc [25].

Women reported their consumption frequency over the past 12 months by choosing from 10 frequency categories ranging from ‘never’ to ‘three or more times per day’. Portion sizes were calibrated using photographs of scaled portions of six foods, and two questions regarding overall daily consumption of fruits and vegetables were used to calibrate consumption of individual fruits and vegetables. The nutritional analysis of the FFQ was conducted independently of the Cancer Council Victoria. The consumption frequency categories were converted to numerical daily equivalent frequencies (e.g., never = 0, once per week = 0.14, once per day = 1, three or more times per day = 3), which were used in conjunction with portion information to estimate the usual amount of food or beverage item consumed as g/day. Usual nutrient intake was then calculated using Australian nutrient databases NUTTAB 2010 [26] and AUSNUT 2007 [27] via FoodWorks 7 (Xyris Software, Queensland, Australia). Dietary data was available for 382 women. Three women who completed the FFQ reported energy intakes >3 SD above the mean, and were excluded from descriptive and inferential dietary analyses [28,29]. No women reported energy intakes <3 SD below the mean.

#### 2.3.3. Supplement Intake

Intake of dietary supplements was self-reported. Participants were asked to record the name of any dietary supplements they were currently using, the frequency of consumption, and the dose. Supplements were characterized as multivitamin/mineral supplements containing iron, multivitamin/mineral supplements containing zinc, iron-specific supplements, or zinc-specific supplements, and the dose of elemental iron or zinc was confirmed by checking supplement packaging or the manufacturers’ websites. To estimate supplemental iron and zinc intake as mg/day, the total mg of iron and zinc from all supplements consumed per week was divided by seven days. Use of supplements containing iron or zinc was also investigated as a dichotomous variable.

#### 2.3.4. Assessing Adequacy of Dietary Intakes

Due to the wide inter-individual variation in menstrual losses, iron requirements in premenopausal women are distributed asymmetrically around the median, thus unlike most nutrients, the cutpoint method of assessing intakes against the Estimated Average Requirement (EAR) for iron (*i.e.*, the amount estimated to meet the requirements of half the healthy individuals in the group) cannot be used to estimate the prevalence of inadequate intakes [30]. The adequacy of dietary iron intakes of the cohort was therefore evaluated using the probability approach [30]. This approach quantifies the prevalence of inadequate iron intakes in a cohort by comparing the cohort’s distribution of usual iron intakes to a proposed distribution of iron requirements [30]. The Institute of Medicine (IOM) [23] has published 14 levels of usual iron intake associated with probabilities of inadequacy for cohorts of women based on oral contraceptive use (cohort not using oral contraception, cohort using oral contraception, and mixed cohort of users and non-users) [23]. As 38% of the women recruited in the present study reported using oral contraception, we used the IOM’s probability values and associated levels of usual intake for a mixed group of oral contraceptive users and non-users. For example, the probability that usual dietary iron intakes of 6.56 to 7.13 mg/day are inadequate is 0.65, whereas the probability that usual intakes ranging from 12.5 to 14.85 mg/day are inadequate is 0.08. The number of participants with usual iron intakes in each of the 14 levels was counted, converted to a percentage of the cohort, and then multiplied by the associated probability value for each level, generating the proportion of women with increased risk of inadequate intakes.

As zinc requirements are assumed to be normally distributed, participants’ risk of inadequate zinc intakes was estimated using the EAR cut-point method [23]. The number of participants with usual dietary zinc intakes below the Australian EAR for zinc for women aged 19–50 years (<6.5 mg/day [31]) was summed and presented as *n* (%).

#### 2.3.5. Biochemistry

In this study, serum ferritin and hemoglobin were used as markers of iron status and serum zinc was used for zinc status. C-reactive protein (CRP) was used to indicate the presence of acute infection or inflammation. For women recruited in Melbourne, fasting venous blood samples were used for all biomarkers, and Dorevitch Pathology (Heidelberg, Melbourne, Australia) conducted analyses of serum ferritin, hemoglobin, and CRP. Serum ferritin was measured using the ADVIA Centaur Ferritin Assay on the Siemens ADVIA Centaur (Siemens Healthcare Diagnostics, Deerfield, Illinois, US), hemoglobin measured using the Sysmex Automated Hematology Analyzer XE-2100 (Sysmex, Kobe, Japan), and CRP measured using a latex-enhanced immunoturbidimetric assay on a Siemens ADVIA 2400 (Bayer Diagnostics, Tarrytown, NY, US). Women recruited at the Blood Service were nonfasting as data collection occurred just prior to making a blood donation, and venous samples were used for analyses except for hemoglobin, which used capillary samples. For women recruited at the Blood Service, the Blood Service conducted analyses of serum ferritin, hemoglobin, and CRP. Serum ferritin was measured using the AxSYM Ferritin assay on the AxSYM (Abbott Diagnostics, Abbott Park, IL, US), fingerprick hemoglobin was measured using the Hemocue B-Haemoglobin Photometer (Hemocue, Angelholm, Sweden), and CRP was measured using the Quantikine Human CRP Immunoassay (R & D Systems, Minneapolis, MN, US).

For measurement of serum zinc concentration, a venous blood sample was collected in a trace element-free vacutainer (cat. no. 368380, BD Australia, North Ryde, Sydney, Australia). For women in Melbourne, sampling took place between 07:40 and 18:30; for women in Sydney, sampling took place between 08:00 and 16:55. Serum was separated and stored at −70 °C in trace element-free microcentrifuge tubes (cat. no. 3013-870-000, Labcon, Ballarat, Australia) prior to transport and analysis for serum zinc in the Trace Element Laboratory at the Department of Human Nutrition, University of Otago, Dunedin, New Zealand. Serum zinc was measured using a ContrAA 700 contiuum source flame atomic absorption spectrometer (Analytik Jena AG, Jena, Germany) following a modified method of Smith *et al.* [32]. Serial replicates of an in-house pooled serum and quality control sera (UTAK, UTAK Laboratories, Valencia CA) were used to check the precision and accuracy of the assay. Samples were analyzed in two batches—those collected from 2010 to 2011 were analyzed in 2011, and those collected from 2012 to 2014 were analyzed in 2014. For the first batch, the interassay CV (as %) for serum zinc was 3.5% (*n* = 17), and the analyzed mean value for the zinc serum quality control was 10.4 μmol/L (CV 5.4%, *n* = 12) compared with the manufacturer’s certified mean (SD) value of 10.4 (2.6) μmol/L. The interassay CV in the second batch was 2.2% (*n* = 6), and the analyzed mean value for the quality control was 10.2 μmol/L (2.4%, *n* = 3) compared with the manufacturer’s certified mean (SD) value of 10.1 (2.4) μmol/L. Serum zinc concentrations were standardized to 08:00 blood sampling using the method of Arsenault *et al.* [33]. Briefly, serum zinc concentrations were standardized using a linear regression model with serum zinc concentration as the dependent variable and blood sampling time in hours as a covariate to obtain the β coefficient for sampling time. Each participant’s sampling time was centered around 08:00 and multiplied by the time coefficient, and this value was then subtracted from each woman’s raw serum zinc concentration. These resulting standardized values were interpreted according to fasting status.

The reference values used to categorize biomarker data are presented in Table 1.

Complete iron and zinc biochemical data were available for 333 women. Seven of these women were excluded from descriptive and inferential analyses of iron and zinc status—one woman who had recently had an intravenous iron infusion (serum ferritin 461 μg/L) and six women who had serum zinc concentrations exceeding 18.97 μmol/L, indicative of potential contamination [34].

**Table 1:** Reference values used to interpret biomarkers of iron and zinc status.

Classification	Biomarker			
	Serum Ferritin	Hemoglobin	CRP	Serum Zinc
Depleted iron stores ^a^	<15 μg/L	≥120 g/L	<5 mg/L	-
Iron overload ^b^	>150 μg/L	-	<5 mg/L	-
Anemia ^a^	-	<120 g/L	-	-
Iron-deficiency anemia ^a^	<15 μg/L	<120 g/L	<5 mg/L	-
Non-iron deficiency anemia	≥15 μg/L	<120 g/L	<5 mg/L	
Low serum zinc ^c^	-	-	-	AM fasting: < 10.7 μmol/L
				AM nonfasting: < 10.1 μmol/L
Inflammation/ infection ^d^	-	-	≥5 mg/L	-

CRP = C-reactive protein. ^a^ World Health Organization/Centers for Disease Control and Prevention [35]. ^b^ World Health Organization [1]. ^c^ Brown, Rivera, Bhutta, Gibson, King, Lonnerdal, Ruel, Sandtrom, Wasantwisut and Hotz [21]. ^d^ Thurnham and McCabe [36].

### 2.4. Statistical Analysis

All statistical analyses were completed using Stata/SE 12.0 (StataCorp, TX, US), and for all tests, the significance level was set at *p* < 0.05.

The normality of continuous variables was assessed by evaluating histograms, with normally distributed data presented as mean (SD) and skewed data presented as median (interquartile range (IQR; 27th to 75th centiles) and geometric mean (95% CI)). Categorical data are presented as *n* (%). To compare groups, independent samples t-tests were used for normally distributed data, Wilcoxon rank-sum tests were used for skewed data, Pearson chi-square tests were as used for categorical data with expected counts ≥5, and Fishers exact tests were used for categorical data with expected counts <5.

Nested linear regression analyses were used to investigate associations between dietary iron intake (mg/day; dependent variable) and dietary zinc intake (mg/day; independent variable), with and without energy intake (MJ/day) as a potential confounding variable. Inspection of residual-versus-fitted and component-plus-residual plots was conducted to confirm adherence to linear regression assumptions, and the fit of the nested models was compared using a likelihood ratio test. DfBeta statistics were produced and the regression analysis was repeated omitting any participants whose DfBeta statistic indicated disproportionate influence on the dietary zinc intake regression coefficient (cut-off DfBeta > 2/√n [37]).

To investigate associations between inadequate dietary iron and zinc intakes, women were dichotomized to those meeting the zinc EAR and those not meeting the zinc EAR, and the percentage of participants at risk of inadequate iron intakes in each group was compared using a two-proportion z-test (an immediate two-sample proportion test).

Nested linear regression analyses were also used to investigate continuous associations between uncorrected serum ferritin (μg/L) as the dependent variable and serum zinc (μmol/L, standardized to 08:00 blood sampling) as the independent variable, with and without potential confounding variables (fasting status, CRP (mg/L), age, BMI, use of oral contraception, mL of blood donated over the past 12 months, use of dietary supplements containing iron and/or zinc). Plotting the residuals against the fitted values showed heteroscedasticity, therefore serum ferritin was natural log transformed for these analyses. The fit of these nested models were also compared using the likelihood ratio test and analysis was repeated omitting women whose DfBeta value indicated undue influence on the serum zinc regression coefficient. Logistic regression was used to investigate whether having depleted iron stores was associated with low serum zinc concentrations, adjusting for the potential confounding variables.

## 3. Results

### 3.1. Characteristics of Participants

Characteristics of study participants are shown in Table 2. Of the 382 women included in the study, 320 women had both dietary data and biochemical data, 59 women had only dietary data, and 3 women had only biochemical data. Eighty-one percent of women were tertiary educated, and 43% had made a blood donation in the previous year. Two-thirds of the cohort had BMI values in the normal range (*n* = 258), and 28% (*n* = 107) were overweight or obese.

**Table 2:** Demographic and anthropometric characteristics of 382 women who participated in the study.

	*N* = 382
Demographics	
Age, years ^a^	28.7 (7.3)
Tertiary education, *n* (%)	307 (81%)
Current smoker, *n* (%)	27 (7%)
Donated blood in previous 12 months, *n* (%)	162 (43%)
Oral contraceptive use, *n* (%)	146 (38%)
On ‘special’ diet, *n* (%)	87 (23%)
Vegetarian, *n* (%)	16 (4.2%)
Mostly vegetarian but eat some meats, *n* (%)	33 (9%)
Other diets, *n* (%) ^b^	38 (10%)
Anthropometrics	
Weight, kg ^a^	66.1 (11.7)
Height, m ^a^	1.66 (0.06)
Body mass index, kg/m^2 a^	23.9 (3.9)

^a^ Mean (SD). ^b^ ‘Other diets’ include gluten-free and/or wheat-free (*n* = 20), vegan (*n* = 5), weight management (including high protein, reduced calories, Lite n Easy, low fat; *n* = 5), Paleolithic/primal (*n* = 2).

### 3.2. Intakes of Iron and Zinc

Participants’ usual intakes of dietary and supplemental iron and zinc are described in Table 3. Average usual dietary iron and zinc intakes were 10.5 mg/day (range 1.8 to 25 mg/day) and 9.3 mg/day (range 1.6 to 40.9 mg/day) respectively. Thirty-one percent of this cohort were at risk of inadequate dietary iron intakes, and 19% were at risk of inadequate dietary zinc intakes. Twenty percent (73/373 women) were taking supplemental iron as either iron-specific or multivitamin/mineral supplements, and three women were taking both iron-specific and multivitamin/mineral supplements. Fourteen percent (52/370) were taking some form of supplemental zinc. Only 2/8 participants taking zinc-specific supplements provided dose information and these supplements contributed 25 to 50 mg/day of elemental zinc. The dose of zinc from multivitamin/mineral supplements ranged from 0.4 to 30 mg/day.

**Table 3:** Dietary and supplemental iron and zinc intakes in women aged 18–50 years (*N* = 379).

	Mean (SD) or *n* (%)	Median (IQR)	Geometric Mean (95% CI)
Dietary iron intake, mg/day	10.5 (3.5)		
At risk of inadequate dietary iron intake, *n* (%) ^a^	117 (31%)		
Iron-specific supplements, *n* (%)	32 (8%)		
Elemental iron, mg/day ^b^		13.3 (5 to 30)	12.1 (6.9 to 21.2)
Vitamin/mineral supplements with iron, *n* (%)	44 (12%)		
Elemental iron, mg/day ^c^		5 (4.9 to 5)	4.6 (3.4 to 6.2)
Dietary zinc intake, mg/day	9.3 (3.8)		
At risk of inadequate dietary zinc intake, *n* (%) ^d^	72 (19%)		
Zinc-specific supplements, *n* (%) ^e^	8 (2%)		
Vitamin/mineral supplements with zinc, *n* (%)	45 (12%)		
Elemental zinc (mg/day) ^f^		6.3 (4 to 10)	5.4 (4.1 to 7.2)

IQR = interquartile range (25th to 75th centiles). ^a^ Assessed using probability approach. ^b^ Supplemental iron dose data unavailable for 5/32 women taking iron-specific supplements. ^c^ Supplemental iron dose data unavailable for 1/44 women taking multivitamin/mineral supplements. ^d^ Assessed by cut-point method using Australian Estimated Average Requirement for women 19–50 years (6.5 mg/day [31]). ^e^ Supplement zinc dose data only available for 2/8 women taking zinc-specific supplements (25 mg/day and 50 mg/day). ^f^ Supplement zinc dose data unavailable for 1/45 women taking multivitamin/mineral supplements.

#### Associations between Usual Dietary Iron and Zinc Intakes

There was a positive association between dietary iron intake and dietary zinc intake. Multiple linear regression indicated that for every 1 mg/day increase of dietary iron intake, mean dietary zinc intake increased by 0.7 mg/day, and 65% of variation in dietary iron and zinc intakes could be accounted for by each other (Table 4). After adjusting for energy intake, each 1 mg/day increase of dietary iron intake was associated with a 0.4 mg/day increase in dietary zinc intake (Table 4). A likelihood ratio test indicated that adjusting for energy intake improved the fit of the model (likelihood ratio χ^2^ = 60.18, *p <* 0.001). Omitting nine women identified using DfBetas as being overly influential observations from the adjusted model did not markedly change results, as a 1 mg/day increase in dietary iron intake still increased mean dietary zinc intake by 0.4 mg/day (95% CI 0.3 to 0.5).

**Table 4:** Multiple regression analysis of associations between dietary iron and zinc intakes in women aged 18–50 years (*N* = 379) ^a^.

Model	β (95% CI) ^b^	*p-*Value
Unadjusted model Adj. *R*^2^ = 0.648, *p <* 0.001	0.739 (0.684 to 0.794)	<0.001
Adjusted model ^c^ Adj. *R*^2^ = 0.730, *p <* 0.001	0.433 (0.359 to 0.507)	<0.001

^a^ In all models, dietary iron intake (mg/day) was the dependent variable and dietary zinc intake (mg/day) was the independent variable. ^b^ Unstandardized β-coefficient. ^c^ Adjusted for energy intake (MJ/day).

Using a two-proportion z-test, the proportion of women at risk of inadequate dietary iron intakes was 63% (95% CI 52 to 74%) among those with dietary zinc intakes below the EAR versus 23% (95% CI 18 to 28%) among those meeting the zinc EAR (*p* < 0.001)

### 3.3. Biochemical Iron and Zinc status

Details of participants’ iron and zinc biomarkers are provided in Table 5. For the 32 women with acute inflammation, serum ferritin was multiplied by a factor of 0.65 [38]. After this correction for inflammation, thirty-seven percent of women had depleted iron stores, of which 18% had iron-deficiency anemia (7% of the total sample). Seventeen percent of the sample (57/326) had low serum zinc concentrations. Chi-square analysis indicated twenty-five percent (31/122) of women using oral contraception had low serum zinc concentrations compared to 13% of nonusers (26/203) χ^2^(1, *N* = 326) = 8.37, *p* = 0.004. Women with fasting samples and women with nonfasting samples did not differ in terms of oral contraceptive use (38% of fasting group using oral contraception *vs.* 37% of nonfasting group) χ^2^(1, *N* = 325) = 0.006, *p* = 0.941.

**Table 5:** Biochemical measures of iron and zinc status in women aged 18–50 years (*N =* 326).

	*n* (%)	Median (IQR) or Mean (SD)	Geometric Mean (95% CI)
Serum ferritin, μg/L ^a^		21 (11 to 38)	19.0 (17.2 to 21.1)
CRP, mg/L		0.63 (0.17 to 2.11)	0.65 (0.55 to 0.77)
Hemoglobin, g/L (*n* = 143)		132 (10)	
Depleted iron stores, *n* (%) ^a,b^	97 (30%)		
Iron-deficiency anemia, *n* (%) ^a,c^	22 (7%)		
Non-iron deficiency anemia, *n* (%) ^d^	7 (2%)		
Iron overload, *n* (%) ^e^	2 (0.6%)		
Elevated CRP, *n* (%) ^f^	32 (10%)		
Fasting serum zinc, μmol/L (*n* = 143) ^g^		12.6 (1.7)	
Low fasting serum zinc, *n* (%)^h^	17 (12%)		
Nonfasting serum zinc, μmol/L (*n* = 183) ^g^		11.8 (2.0)	
Low nonfasting serum zinc, *n* (%)^h^	40 (22%)		

IQR = interquartile range (25th centile to 50th centile). CRP = C-reactive protein. ^a^ Serum ferritin corrected for acute infection: In women with CRP ≥5 mg/L was multiplied by a factor of 0.65 as suggested by Thurnham [38]. ^b^ Depleted iron stores defined as corrected serum ferritin <15 μg/L, hemoglobin ≥120 g/L [35]. ^c^ Iron-deficiency anemia defined as corrected serum ferritin <15 μg/L, hemoglobin <120 g/L. ^d^ Non-iron deficiency anemia defined as hemoglobin < 120 g/L and corrected serum ferritin ≥15 μg/L [35]. ^e^ Iron overload defined as serum ferritin >150 μg/L and CRP <5 mg/L [1]. ^f^ Elevated CRP defined as ≥5 mg/L [36]. ^g^ Serum zinc standardized to 08:00 sampling time. ^h^ Low serum zinc defined according to fasting status—fasting: <10.7 μmol/L; nonfasting: <10.1 μmol/L [21].

#### Associations between Iron and Zinc Status

There was a small positive association between serum ferritin and serum zinc concentrations. Simple linear regression of natural log transformed serum ferritin (not corrected for acute infection) on serum zinc indicated that an increase of 1 μmol/L in serum zinc was associated with a 10% increase in mean serum ferritin (Table 6). This association decreased slightly after adjusting for fasting status, CRP, age, BMI, use of oral contraception, blood donation, and use of iron and/or zinc supplements, so that an increase of 1 μmol/L in serum zinc was associated with a 6.4% increase in mean serum ferritin (Table 6). A greater proportion of the variance was explained by the adjusted model compared to the unadjusted model, and a likelihood ratio test indicated that the fit of the adjusted model was better than that of the unadjusted model (likelihood ratio χ^2^ = 60.47, *p <* 0.001).

A more conservative model omitting 16 women identified as having disproportionate influence on the serum zinc regression coefficient did not change the findings, with 1 μmol/L in serum zinc associated with a 5.7% increase in mean serum ferritin (antilogarithm β 95% CI 1.004 to 1.11).

**Table 6:** Multiple regression analysis of associations between serum ferritin and serum zinc concentrations in women aged 18–50 years (*N =* 326) ^a^.

	Logarithmic Scale β (95% CI) ^b^	Antilogarithm of β (95% CI)	*p-*Value
Unadjusted model Adj. *R*^2^ = 0.035, *p <* 0.001	0.096 (0.043 to 0.148)	1.100 (1.043 to 1.160)	<0.001
Adjusted model ^c^ Adj. *R*^2^ = 0.181, *p <* 0.001	0.061 (0.010 to 0.112)	1.063 (1.010 to 1.118)	0.019

^a^ In all models, natural log transformed μg/L serum ferritin was the dependent variable and untransformed serum zinc (μmol/L, standardized to 08:00 sampling) was the independent variable. ^b^ Unstandardized β coefficients. ^c^ Adjusted for fasting status, C-reactive protein (mg/L), age (years), BMI (kg/m^2^), oral contraceptive use, blood donation (mL/year), use of dietary supplements containing iron and/or zinc.

No association was observed between low serum ferritin and low zinc levels in this cohort. Fifteen percent (32/213) of women with adequate iron stores presented with low serum zinc concentration, compared to 22% (25/113) of women with depleted iron stores, however these proportions were not statistically different χ^2^(1, *N* = 326) = 2.58, *p* = 0.108. Furthermore, using logistic regression to account for fasting status, CRP, oral contraceptive use, and supplement use found women with low serum zinc concentrations were not at increased risk of depleted iron stores compared to women with normal serum zinc concentrations (odds ratio for having depleted iron stores if having low serum zinc = 1.37, *p* = 0.340 (95% CI 0.72 to 2.64)).

## 4. Discussion

We found a significant positive relationship between usual dietary iron intakes and dietary zinc intakes in our cohort of Australian premenopausal women. Each 1 mg/day increase in dietary iron intake corresponded with a 0.4 mg/day increase in dietary zinc intake. Furthermore, women with dietary zinc intakes below Australian recommendations (EAR of 6.5 mg/day [31]) were at greater risk of inadequate dietary iron intakes as determined using the probability approach (63% at risk *vs.* 23% at risk). Our findings support observations in other cohorts that food sources of zinc are also important food sources of iron [6,7], and also supports the theory that usual low intakes of either mineral increase the likelihood of low intakes of the other [8].

We also observed a small positive association between biochemical iron and zinc status, with a 1 μmol/L increase in serum zinc corresponding to a six percent increase in serum ferritin. This finding is in line with previous research that also reported a positive association [14,16,17], however in our cohort, women with biomarkers below internationally-recognized cut-offs for low levels of one mineral were not more likely to present with low levels of the other mineral. Together, these findings suggest that while there may be a positive relationship between iron and zinc status at a population level, it may be too small to translate to a clinically meaningful association among women in Australia. Serum ferritin is not a useful proxy for serum zinc concentrations in our study setting, and women with depleted iron stores are not at increased risk of impaired zinc status. Given our findings that 17% of our cohort are at risk of zinc deficiency and given the health consequences associated with impaired zinc status [19,20], further work is needed to determine the zinc status of women in the Australian population.

Our observation of a strong association between usual iron and zinc intakes was not accompanied by a similarly strong association between iron and zinc status, possibly reflecting the nutrients’ different responses to potential inhibitors and enhancers of absorption. Prosser *et al.* investigated the effect of a four-month dietary iron intervention on serum zinc of premenopausal women [17]. Women with low iron stores were randomized to a group receiving 50 mg/day supplemental iron, a placebo group, or a whole-diet intervention designed to increase iron bioavailability [39]. Women in the dietary intervention did not change their dietary iron or dietary zinc intakes, but their intakes of meat/fish/poultry, heme iron, and vitamin C increased and phytate intakes decreased [17,39]. This diet improved serum ferritin by 26% [39], however it had no effect on serum zinc concentrations compared to the placebo group [17]. An increase in serum ferritin concentration via dietary means is not necessarily partnered with an increase in serum zinc concentration, perhaps as factors influencing the proportion of iron available for absorption are not as influential on the bioavailability of zinc, and an increase in absolute zinc intake might also be needed to increase serum zinc in women. Although we found a strong association between dietary iron and zinc intakes, it seems targeted advice specific for each nutrient is required, and bioavailability of both cannot be improved through the same dietary means.

In contrast to previous research investigating both iron status and zinc status of women in Western countries [14,15,16], we incorporated an acute inflammatory marker in our analyses. Using a regression model that included CRP only slightly attenuated the association between iron and zinc status, perhaps due to the small proportion of women with elevated CRP in our sample (10%). In other groups, measuring and correcting for CRP may be prudent if investigating a relationship between iron and zinc status. The distribution of CRP concentrations in the present sample falls below the distribution observed among women from the National Health and Nutrition Examination Survey (NHANES) 1999–2000 aged 20–49 years (median CRP approximately 2 mg/L (IQR 0.8 to 6 mg/L)) [40]. In this NHANES cohort, at least 25% of women had CRP > 5 mg/L, and in such groups who are more prone to inflammation and infection, correcting for CRP may be more important. Where risk of inflammation is unknown, we suggest erring on the side of measuring CRP.

Our investigation supports findings from previous research suggesting a small statistical association between iron and zinc status in premenopausal women [14,15,16,17]. The strengths of this present study was use of internationally recognized biomarkers for iron status and zinc status, and use of conservative statistical analyses. Limitations include the sample of participants perhaps not being representative of premenopausal women in Australia (e.g., high proportion of women with tertiary education [41], majority with BMI values within the normal range [42], lower proportion of current smokers [42]) and use of a FFQ to assess adequacy of usual dietary iron and zinc intakes [43], although correlations for dietary iron intake in the previous version of our FFQ were comparable to that found in the validation of the Block FFQ against two four-day diet records (*r* = 0.34 to 0.42 [44]) and the validation of the Willett FFQ against a one-year diet record (*r* = 0.47 [43]) (validation of these two FFQs for dietary zinc intakes has not been published). We acknowledge that the IOM values used to conduct the probability method of assessing usual dietary iron intake may not represent the iron requirements of our cohort as there was a greater prevalence of oral contraceptive use among our participants than proposed by the IOM, and the IOM values do not account for blood donation. Our assessment of risk of inadequacy associated with dietary intakes must therefore be interpreted with caution. The use of oral contraception may also inflate our prevalence of low serum zinc concentrations, as oral contraceptives are known to suppress serum zinc concentration via hemodilution [21]. In addition, the present analysis of associations between iron and zinc status did not account for menstrual blood loss, a known influence in iron status [45]. To confirm the findings of our study, population-based studies assessing biochemical iron and zinc status and using rigorous dietary assessment methods are required, as is information on how current rates of oral contraceptive use and blood donation might affect women’s iron and zinc requirements.

## 5. Conclusions

In conclusion, we found positive associations between usual dietary iron and zinc intakes in premenopausal women, with those meeting dietary zinc recommendations being less likely to have inadequate iron intakes compared to those not meeting zinc recommendations. We also found a small positive association between iron status and zinc status in the same cohort, although this association may not be clinically meaningful. Our data do not support the ability to identify women at risk of impaired zinc status through exploration of iron status, and further work understanding the zinc status of women within the Australian population is required.
